# Data Sources for Improving Estimates of the Global Burden of Injuries: Call for Contributors

**DOI:** 10.1371/journal.pmed.1000001

**Published:** 2009-01-20

**Authors:** Kavi Bhalla, James Harrison, Jerry Abraham, Nagesh N Borse, Ronan Lyons, Soufiane Boufous, Limor Aharonson-Daniel, on behalf of the Global Burden of Disease Injury Expert Group

## Abstract

Kavi Bhalla and colleagues invite individuals and organizations to provide local injury data sources to help inform estimates of the global burden of injuries.

We have recently embarked on a collaborative project to improve estimates of the global burden of injuries. This commentary invites individuals and organizations to contribute to building this global public good by providing the project access to relevant results from local injury data sources.

Reliable estimates of the incidence and burden of injuries are essential inputs for prioritizing prevention strategies. While population-based injury surveillance systems are obviously the best source for such information, it is also widely recognized that such infrastructure is unlikely to be established in most of the world for several decades. Thus, there is an urgent need for the global injury community to collaborate to build the methods and tools that can be used to derive reasonable estimates from a wide range of existing sources, including hospital records, police reports, health surveys, and death registers, among many others.

On July 14, 2007, the core team of the Global Burden of Disease (GBD) project issued an open call for expert participation to ensure that GBD 2005 estimates incorporate the broadest possible knowledge of individual diseases and risk factors [1]. The original GBD study [2] has proven immensely influential in shaping global health priorities, and many in the injury community recognize the need and potential of replicating such work with a focus on injury metrics. As a result, over 160 injury researchers from across the globe volunteered to participate in the GBD Injury Expert Group. The mandate of the group is to further develop the theoretical framework for estimating the burden of injuries, as well as to collect and analyze all available regional data sources. While keeping in mind the need to summarize best evidence for GBD 2005, the expert group also aims at developing these methods for similar work in the future. Various aspects of the group's efforts can be tracked online at our public Web site [3].

## Challenges in Estimating the Global Burden of Injuries

A basic guiding principle for this project is that best estimates of the burden of deaths and nonfatal injuries should be generated by inclusion of all existing knowledge and sources of information. Thus, arguably, the most important inputs for this project are existing local, regional, and national administrative data sources that routinely collect information about victims as well as research studies that have collected relevant information as part of their investigation. It is important to note that most countries do not have injury surveillance systems capable of directly reporting population-level estimates of deaths and nonfatal injuries. Thus, analytical tools are needed to maximize the utility of all known data sources for estimating the burden of injuries (see, for instance, [3,4]). In the context of estimating the global burden of injuries, relevant data sources (Box 1) can be used for improving the estimates of incidence of injuries (e.g., by external cause: road traffic crashes, falls, fires, etc.), correlating external causes with injury outcomes (e.g., likelihood of hip fractures in road traffic crashes), and/or for improving the estimates of the health burden associated with the disability due to nonfatal injuries. Few data sources can inform all three aspects. For instance, while population health surveys can help estimate incidence of injuries, they rarely provide reliable information about injury sequelae. Similarly, while medical records are one of the best sources for injury diagnosis, they are often not population representative. Thus, piecing together information from multiple sources is a necessary aspect of estimating the burden of injuries.

**Box 1. What Are Relevant Data Sources and Variables?**

**1. Data sources for estimating deaths:** High-quality cause-of-death registration data are the gold standard. In their absence, we can estimate from incomplete or subnational death registration, mortuary records, funeral/cemetery records, and police reports.

**2. Data sources for estimating burden of nonfatal events:**

- Health surveys: Population-representative health surveys that include questions on injuries are important sources for estimating incidence of nonfatal injuries. Surveys that disaggregate by type of care are particularly useful.
- Hospital registries: Hospital records provide International Classification of Diseases–coded injury diagnosis, which is needed for estimating public health burden. We are particularly interested in registries that include both external causes and injury diagnosis. Registers of admitted patients and registers of patients attending emergency departments are both valuable.

**3. Variables of interest:** This project is primarily interested in the injured person's age, sex, external cause of injury, and injury diagnosis. We are also interested in variables that enable best use of the data to estimate population incidence of injury (e.g., variables that allow control of double counting of cases).

**4. Time period of interest:** GBD 2005 will generate estimates for the years 1990 and 2005. However, when data from these years are not available, the project will analyze information from the closest time period for which data are available.

**5. Characteristics of good data sources:**

- For estimating incidence: High coverage/completeness or population representativeness; few cases assigned to unspecified categories.
- For estimating burden of nonfatal injuries: medically certified diagnosis (e.g., International Classification of Diseases–coded hospital records); inclusion of both external causes and injury outcomes; follow-up studies to estimate long- term disability consequences.

Harmonizing results from these diverse sources of information requires the expert group to address a range of difficult analytical and theoretical issues. These include: reclassification of cases assigned to missing or unspecified categories, estimation of the burden associated with multiple trauma, the development of mappings from various external causes to injury outcomes, and the estimation of population incidence of injuries by coupling hospital administrative records with household surveys. These and various other issues pertinent to the estimation of the burden of injuries are being examined by expert group members and are being documented in a series of theoretical papers as described on our Web site [3]. While addressing the theoretical issues and building such a toolkit is a complex task, the expertise in the existing membership of our expert group and past work by the broader injury community [4] will likely result in progress in this direction. However, getting access to the data sources on which these tools will be applied is a much bigger hurdle.

## Call for Contributors

**Figure 1:**
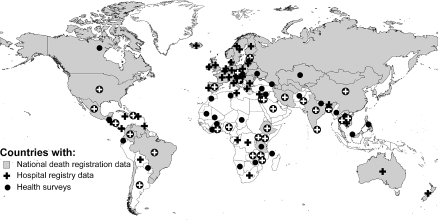
Known Data Sources that Could Contribute to the GBD Injuries Estimates If Data Access Were Available This continuing environmental scan can be tracked on the expert group Web site [3].

The expert group has been conducting an environmental scan to identify all potential sources of information for such work. Figure 1 shows that data sources that can inform such estimates exist in all regions of the world, including regions such as sub-Saharan Africa that have been traditionally considered information poor. It is essential that most, if not all, of these sources inform this project. The group urges all those who support the objectives of the project to help us get access to these information sources (Box 2). The project will evaluate all potential data sources for quality and wherever possible put them to appropriate use.

**Box 2. How Can You Participate?**

**You can contribute data by:**

1. Adding to the ongoing environmental scan: Tell us about other relevant data sources that potentially contain variables of interest to the project.

2. Helping us get access to data sources listed in the environmental scan. Data can be shared with our project in any of the following ways:

- Provide us with case-level records. Availability of unit-level records (microdata) allows us to use advanced analytical tools to correct for data quality (e.g., reassignment of ill-defined cause categories).
- Run our data extraction scripts and send us tabulations: We can provide scripts for most commonly used statistical packages that would produce appropriately grouped tabulations of the relevant variables.
- Provide us with any reports or publications that tabulate the relevant variables, and we will use algebraic mappings to the GBD definitions.

We request permission to share data sets with other researchers but will respect all data access restrictions requested by the owners of the information.

**Funding for data collection:** Although we do not have funding to support new data collection, we can work with groups to raise funds needed for access and translation of existing data sources.

**Joining the expert group:** The GBD Injury Expert Group is comprised solely of volunteer members who share a common interest in improving injury metrics from existing data sources. Although our work is unpaid, many of us have existing funding in closely related areas. We expect that group members will produce many collaborative research publications.

It is important to understand that in most regions of the world, there are no existing data readily available to the project. In the absence of local data sources, the GBD project will estimate the burden of disease and injury using extrapolation models. If we want to see sensible estimates of injuries produced in this influential study, the burden of providing the best evidence as input rests upon the entire international health community.

**For More Information:**

**Visit:** http://sites.google.com/site/gbdinjuryexpertgroup/

**Contact:**

James Harrison, Research Center for Injury Studies, Flinders University, Adelaide, South, Australia, 5001, Australia, Phone: +61 8 8201 7602, Fax: +61 8 8374 0702, E-mail: james.harrison@flinders.edu.au

Kavi Bhalla, Harvard Initiative for Global Health, Cambridge, Massachusetts, 02138, United States of America, Phone: +1 617 496 6903, Fax: +1 617 495 8231, E-mail: kavi_bhalla@harvard.edu

## Abbreviations

GBD: Global Burden of Disease
